# AUF1 is involved in splenic follicular B cell maintenance

**DOI:** 10.1186/1471-2172-11-1

**Published:** 2010-01-11

**Authors:** Navid Sadri, Jin-Yu Lu, Michelle L Badura, Robert J Schneider

**Affiliations:** 1 Department of Microbiology, New York University School of Medicine, 550 First Avenue, New York, NY 10016, USA; 2 BCMM 231, Howard Hughes Medical Institute, Yale University School of Medicine, 295 Congress Ave, New Haven, CT 06536, USA

## Abstract

**Background:**

The adenosine/uridine-rich element (ARE)-binding protein AUF1 functions to regulate the inflammatory response through the targeted degradation of cytokine and other mRNAs that contain specific AREs in their 3' noncoding region (3' NCR). To investigate the role of AUF1 in the immune system, we characterized the lymphoid compartments of AUF1-deficient mice.

**Results:**

Mice lacking AUF1 exhibit an altered proportion and size of splenic B cell subsets. We show prominent apoptosis in splenic B cell follicles and reduced expression of Bcl-2, A1, and Bcl-X_L _correlate with increased turnover and significant reduction in the number and proportion of splenic FO B cells in AUF1-deficient mice. In addition, AUF1-deficient mice exhibit a sharp decrease in splenic size and lymphocyte cellularity. Bone marrow transfer studies demonstrate that AUF1 deficiency induces cell-autonomous defects in mature B cell subsets but not in the overall number of splenocytes. Reconstitution of irradiated adult AUF1-deficient mice with wild-type bone marrow restores the proportion of FO and marginal zone (MZ) B cells, but does not rescue the decrease in the number of splenocytes. Functionally, AUF1-deficient mice mount an attenuated response to T cell-independent (TI) antigen, which correlates with impaired MZ B cell function.

**Conclusion:**

These data indicate that AUF1 is important in the maintenance of splenic FO B cells and adequate humoral immune responses.

## Background

The mammalian spleen functions to remove old and damaged erythrocytes, participates in the immune response, particularly against blood-borne pathogens, and is the major site for peripheral B cell development [1]. Immature surface immunoglobulin-expressing B cells that reach the spleen from the bone marrow (referred to as 'transitional' B cells) represent developmental precursors to mature follicular (FO) B lymphocytes, the major mature B cell population in the spleen [2,3]. The other two mature B cell populations consist of non-circulating splenic marginal zone (MZ) B cells that are believed to be derived from transitional cells, and B-1 cells, that are controversial in origin and are enriched in peritoneal and pleural cavities [4]. FO B cells contribute to most T-cell dependent (TD) responses that induce germinal center (GC) development of affinity-matured long lived plasma cells and memory B cells [4,5]. In contrast, MZ cells and B-1 cells are predominately responsible for the initial rapid T-cell independent (TI) IgM antibody response and form an important line of defense against antigens and pathogens in the blood and mucosal sites [4,6]. More recent analysis of B cell populations suggests that there exist two distinguishable, long-lived, recirculating post-transitional follicular B cell populations, the mature FO B cell subset described above and a new subset referred to as FO-II B cells. The FO-II B cells differ from FO B cells in that they develop in an antigen-independent manner and may serve as a follicular precursor to both MZ B cells and FO B cells [7].

Although the precise mechanisms are still unclear, maintenance of each peripheral B cell subset is affected by the availability of resources, the local environment, and interactions with other cell types [8]. It is known that signaling through the B cell receptor (BCR) is required for the development and maintenance of mature splenic B cells [9]. Genetic studies utilizing knockout mouse models for various BCR complex components and downstream effectors clearly demonstrate that an adequate BCR signal is indispensable for the development of transitional B cells and further differentiation into mature B cells [2,10-14]. Furthermore, an ongoing or 'tonic' BCR signal is required for B cells to survive [11,15]. This was elegantly shown using an inducible disruption of the BCR in mature B cells, which resulted in the absence of all three mature subsets [16]. In addition to BCR signaling, both the splenic microenvironment and the ability to respond to locally produced growth factors play equally important roles in the development and maintenance of mature B cells [17,18].

Many short-lived mRNAs that encode cytokines and cell survival factors contain an adenosine/uridine-rich element (ARE) in the 3' non-coding region that allows for their post-transcriptional regulation by ARE-binding proteins. AUF1, also known as heterogeneous nuclear ribonucleoprotein D, is important in promoting the decay of ARE-containing mRNAs [19-21]. In addition to mRNA turnover, AUF1 has more recently been implicated in a number of other cellular processes, including mRNA translation [22] and chromatin remodeling [23]. The physiological role of AUF1 in controlling the decay of key pro-inflammatory cytokine mRNAs, interleukin (IL)-1β and tumor necrosis factor (TNF)-α, was demonstrated by the increased sensitivity of AUF1^-/- ^mice to endotoxic shock and lethality [20]. AUF1 also regulates inflammation within the skin, and AUF1^-/- ^mice develop atopic-like dermatitis [24].

Given the high expression of AUF1 in lymphoid organs [25], we characterized the lymphoid compartment of AUF1-deficient mice. Here we show that AUF1^-/- ^mice develop spleens of reduced size that support a roughly two-fold reduction in the number of lymphocytes. FO B cells exhibit a two-fold decrease in average half-life in AUF1^-/- ^mice that correlates with reduced expression of Bcl-2, A1, and Bcl-X_L_, as well as prominent apoptosis within splenic follicles. The increased turnover of FO B cells corresponds to their decreased frequency in AUF1^-/- ^mice. Our studies indicate that AUF1 plays an important role in regulating splenic lymphocyte cellularity and FO B cell maintenance.

## Methods

### Mice

The AUF1^-/- ^mice were derived as previously described [20]. RAG1^-/- ^mice (B6.129S7-Rag1^tm1Mom^) and CD45.1 mice (B6.SJL-*Ptprc^a^Pep3^b^*/BoyJ) were purchased from Jackson Labs. For adoptive transfer studies, 5 × 10^6 ^donor-derived bone marrow cells were injected intravenously into sub-lethally irradiated (500 rad) RAG1^-/- ^or into lethally irradiated (950 rad) AUF1^+/+ ^and AUF1^-/- ^recipient mice. Chimera mice were analyzed 10 weeks post transfer. CD45 allele expression was used to distinguish donor and recipient populations. All mice are kept under specific pathogen-free conditions. All animal protocols were approved by the NYU Institutional Animal Care and Use Committee.

### Flow Cytometry

Single cell suspensions were prepared from spleen, inguinal and mesenteric lymph nodes, peritoneum lavage, bone marrow, and thymus. Cells were stained and analyzed as previously described [20]. Cell sorting was performed on a BD FACSVantage cell sorter. The following Abs were used: from BD PharMingen: anti-B220-PerCP (RA3-6B2), anti-CD3-PerCP (17A2), anti-CD4-FITC (GK1.5), anti-CD8-APC (53-6.72), anti-IgM-FITC (R6-60.2), anti-CD94-APC (18d3), Streptavidin-APC, Streptavidin-FITC; from Caltag: anti-CD62L-PE (MEL-14), anti-CD23-PE (B3B4); from eBioScience: anti-CD44-biotin (IM7), anti-IgM-biotin (II/41), anti-CD5-FITC (53-7.3), anti-CD24-PE (M1/69), anti-IgD-biotin (11-26), anti-CD21/CD35-FITC,-APC (4E3), anti-F4/80-biotin (BM8), anti-CD86-biotin (GL1), anti-CD45.1-biotin (A20), and anti-CD45.2-biotin (104). Bcl-2 levels were detected using the Bcl-2 kit (BD Pharmingen), following manufacturer's instructions. The set contains anti-Bcl-2-PE (3F11) and PE-conjugated isotype control.

### Immunohistochemistry

Hematoxylin and eosin stained sections were processed as previously described [20]. Germinal center staining was performed at 8 and 22 days post-immunization with TNP-Ficoll and TNP-KLH, respectively. The reagents used were from eBioscience: anti-mouse B220 (RA3-6B2); Cell Signaling: rabbit anti-cleaved caspase-3 (5A1); BD Pharmigen: CR1 (8C12); Vector Laboratory: biotinylated peanut agglutinin, biotinylated anti-rabbit IgG, Vectastain ABC-AP, Vectastain ABC, Vector blue AP kit, and Vector NovaRED peroxidase kit.

### BrdU labeling

Mice were injected intraperitoneally with 1 mg of BrdU (Sigma-Aldrich) and fed with drinking water containing 1 mg/ml BrdU for 7 days. At 0, 7, and 21 days post BrdU administration, spleens from 3 mice per group were analyzed by FACS. Cells were stained with anti-CD21-FITC and anti-CD24-PE or anti-CD23-PE, and then were fixed for 16 h at 4°C with 3.4% paraformaldehyde and 0.01% Tween-20 in PBS. Cells were washed, incubated for 30 min at 37°C with 5 mM MgCl_2_, 1% BSA, and DNase I (100U) in PBS, then labeled with biotinylated anti-BrdU (Br-3, Caltag) or biotinylated anti-IgG control (eBioscience), and subsequently stained with Streptavidin-APC. The mean BrdU labeling at 7 days was used to calculate the labeling rate per day; 2.5% and 1.5% per day in KO and WT mice, respectively. To calculate average life span, the mean values were plotted as a function of time in the chase period, and linear regression analysis was performed to calculate time to lose the BrdU labeled population, assuming a constant pool size during the analysis.

### B cell isolation, Ca^2+ ^mobilization response, in vitro proliferation and survival assays

Splenic B cells were purified with the MACS CD45R microbead system and were verified by FACS to be ≥ 98% B220^+^. Splenic B cells or sorted MZ B cells were loaded with 2 μM Fluo-4/AM (Molecular Probes), stimulated with 10 μg/ml F(ab')_2 _goat anti-IgM Ab and Ca^2+ ^mobilization was recorded on live gated cells. For proliferation assays, 2 × 10^4 ^MZ B cells were plated in triplicate in 96 well plates and stimulated with 10 μg/ml F(ab')_2 _goat anti-IgM Ab or with 10 μg/ml LPS. For the last 8 h of the 48 h culture period the cultures were pulsed with 1 μCi/well [^3^H]-thymidine. Incorporated radioactivity was quantified by scintillation counting. For CD86 up-regulation studies, CD86 expression was analyzed by flow cytometry before and after 24 h treatment with F(ab')_2 _goat anti-IgM Ab (10 μg/ml). For cell viability assays, sorted FO B cells were cultured as stated and then analyzed by flow cytometry after the addition of 7-amino-actinomycin (7-AAD) (5 μg/ml).

### Quantitative real-time RT-PCR

FACS-sorted splenic B220^+^CD21^int^CD24^lo ^FO B cells were homogenized in Trizol (Invitrogen) and mRNA was extracted according to manufacturer's instructions. Bcl-2, A1, and Bcl-X_L _mRNA levels were determined using the Roche LightCycler system. C_T _values were used to calculate relative values and were normalized to C_T _values for cyclophilin A. The following primer pairs were used:

Bcl-2, CCTGTGGATGACTGAGTACC and GAGACAGCCAGGAGAAAT

A1, CTTCAGTATGTGCTACAGGTACCCG and TGGAAACTTGTTTGTAAGCACGTCCAT

Bcl-X_L_, AGAAGAAACTGAAGCAGAG and TCCGACTCACCAATACCTGCGTCCAT

BAFF, CTGTGGTCACTTACTCCAAAGG and GGATCAGATTCAACGGGTCACG

BAFF-R, GCCCAGACTCGGAACTGTCCCA and GCCCAGTAGAGATCCCTGGGTTCC

CypA, TATCTGCACTGCTAAGACTGAATG and CTTCTTGCTGGTCTTGCCATTCC.

### Immunoblot analysis

Immunoblot analysis was performed according to standard protocols using ECL detection (PerkinElmer). Polyclonal antibodies to AUF1 (995) and to eIF4E (Sigma) were used.

### Humoral response assay

Total pre-immune serum and TNP-specific titers were determined using the SBA Clonotyping ELISA kit from SouthernBiotech according to manufacturer instructions. To evaluate TI-II responses, mice were immunized with 10 μg TNP-Ficoll (Biosearch Technologies) and bled 7 days post immunization. To evaluate TD responses, mice were immunized with 20 μg TNP-KLH (Biosearch Technologies) with adjuvant (Imject Alum, Pierce) and bled 14 days post immunization. For *in vitro *studies, 10^6 ^purified B cells were stimulated with murine IL-4 (20 ng/ml) and LPS (25 μg/ml) for 96 h. Immunoglobulin secretion in the supernatant was measured as described in immunization studies. RT-PCR looking for post-switch transcripts was performed as previously described [26].

### Statistical analysis

Data are presented as mean ± standard deviation. For statistical comparison of two samples, the two-tailed Student *t*-test was used for evaluation.

## Results

### Reduction of FO B cells in spleens of AUF1-deficient mice

**Figure 1:**
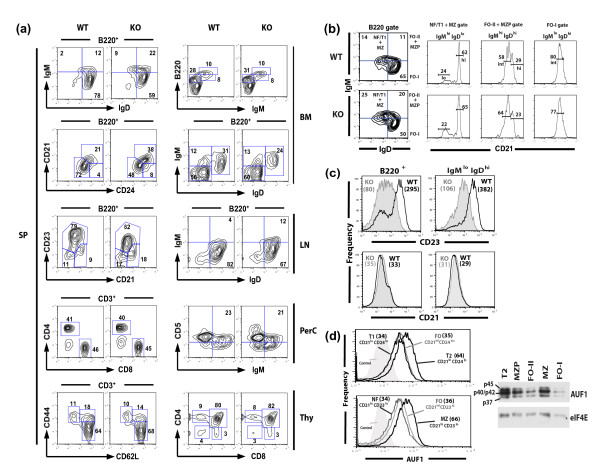
**Flow cytometric analysis of lymphocyte populations in AUF1^-/- ^mice**. (a) Single cell suspensions from spleen (SP), bone marrow (BM), lymph node (LN), peritoneal cavity (PerC), and thymus (Thy) of AUF1^-/- ^(KO) and wild-type (WT) littermates were stained with the indicated antibodies and analyzed. Numbers indicate the percentage of lymphoid cells in quadrants or enclosed within indicated gates. Contour plots gated for B220^+ ^or CD3^+ ^lymphocytes are indicated. Note decrease in FO B cells in AUF1^-/- ^spleens as represented by IgD^hi^IgM^lo^, CD21^int^CD24^lo^, or CD21^int^CD23^hi ^gates. There is also an increase in the proportion of MZ B cells in KO spleens as represented by CD21^hi^CD23^lo ^gate. (b) Splenic B cells were analyzed for IgD and IgM expression (*left panels*) and gated populations were then analyzed for CD21 expression (*right panels*). This analysis distinguishes five different splenic populations (from left to right): NF, IgM^high^IgD^low^CD21^low^; MZ, IgM^high^IgD^low^CD21^high^; FO-II, IgM^high^IgD^high^CD21^int^; MZP, IgM^high^IgD^high^CD21^high^; and FO-I, IgM^low^IgD^high^CD21^int^. Three mice were analyzed in each group. (c) Decreased CD23 expression in AUF1^-/- ^B cells (B220^+^) and FO B cells (IgM^lo^IgD^hi^) from AUF1^-/- ^mice. (d) AUF1 expression in B cell subsets as determined by FACS (*left panels*) or immunoblot analysis (*right panels*). In immunoblot analysis, the staining with AUF1 shows three bands corresponding to the four isoforms of AUF1, and level of eIF4E is shown as a control. Numbers in parenthesis in (c) and (d) depict mean fluorescence intensity (MFI).

AUF1^-/- ^mice were found to have 1.5-fold smaller spleens (71.3 ± 9.4 *vs*. 52.6 ± 3.1 mg) and a roughly two-fold reduction in the number of splenocytes as compared to wild-type controls (Table 1). Splenocyte reduction was restricted to B and T lymphocytes, as the number of F4/80^+ ^macrophages, CD94^+^CD3^- ^natural killer, and CD16/32^+^B220^- ^cell populations in the spleens of AUF1^-/- ^mice were unaffected (Table 1). Except for a decrease in mature IgM^lo^IgD^hi ^B cells, AUF1^-/- ^mice exhibited no significant differences in the cell populations of inguinal and mesenteric lymph nodes, thymus, bone marrow or peritoneal cells (Figure 1a; Table 1), as compared to wild-type littermates. The unaltered cell subpopulations of the bone marrow and thymus in AUF1^-/- ^mice suggest that the reduction in splenocytes in these mice occurred at the periphery within the spleen rather than during development.

**Table 1:** Splenocyte subpopulations in AUF1^+/+^, AUF1^-/- ^and chimeric mice.

	AUF1^+/+^ (*n *= 10)	AUF1^-/-^ (*n *= 10)	WT→RAG1^-/-^ (*n *= 4)	KO→RAG1^-/-^ (*n *= 4)	WT→KO (*n *= 4)	KO→WT (*n *= 4)
Total splenocytes	70.8 ± 10.7	41.6 ± 7.6	34.8 ± 3.6	24.7 ± 3.8	47.3 ± 4.1	73.6 ± 7.9
						
B220^+^	37.5 ± 3.3	23.4 ± 1.5	16.6 ± 0.9	10.6 ± 1.4	24.3 ± 6.2	39.7 ± 4.3
**IgM^lo^, IgD^hi ^(FO)**	26.0 ± 2.3 **(69.3)**	13.1 ± 0.8 **(55.3)**	11.6 ± 0.1 **(69.6)**	5.3 ± 0.7 **(50.0)**	16.7 ± 0.6 **(68.8)**	21.0 ± 1.7 **(53.0)**
IgM^hi^, IgD^hi ^(T2)	4.6 ± 0.9 (12.3)	4.3 ± 0.5 (18.2)	1.5 ± 0.3 (9.3)	1.6 ± 0.5 (14.6)	3.1 ± 0.9 (12.8)	8.2 ± 0.2 (20.7)
IgM^hi^, IgD^lo ^(T1)	3.1 ± 0.5 (8.3)	2.4 ± 0.3 (9.8)	0.9 ± 0.2 (5.2)	1.1 ± 0.1 (10.5)	1.3 ± 0.1 (5.2)	3.9 ± 1.1 (9.9)
						
CD21^hi^CD23^lo ^(MZB)	3.6 ± 0.6 (9.6)	4.3 ± 0.5 (18.4)	2.2 ± 0.2 (13.2)	2.7 ± 0.3 (25.7)	1.6 ± 0.4 (6.6)	5.2 ± 1.2 (13.1)
**CD21^int^CD23^hi ^(FO)**	29.8 ± 0.8 **(79.5)**	13.2 ± 2.7 **(59.1)**	11.6 ± 1.9 **(70.0)**	5.5 ± 0.9 **(52.0)**	20.6 ± 0.2 **(84.9)**	23.9 ± 1.3 **(60.2)**
CD21^lo^CD23^lo ^(NF)	3.4 ± 0.7 (9.1)	3.9 ± 2.6 (17.5)	2.5 ± 0.2 (15.1)	2.2 ± 0.9 (20.5)	1.9 ± 0.5 (7.8)	10.0 ± 0.1 (25.3)
						
IgM^hi^IgD^lo^CD21^lo ^(NF)	2.0 ± 0.3 (5.2)	2.3 ± 0.3 (8.0)	ND	ND	ND	ND
IgM^hi^IgD^hi^CD21^int ^(FOII)	3.4 ± 0.6 (9.1)	3.6 ± 0.8 (13.1)	ND	ND	ND	ND
**IgM^lo^IgD^hi^CD21^int ^(FO)**	24.3 ± 2.4 **(66.0)**	14.6 ± 2.0 **(53.0)**	ND	ND	ND	ND
IgM^hi^IgD^hi^CD21^hi ^(MZP)	1.7 ± 0.4 (4.5)	1.5 ± 0.3 (5.5)	ND	ND	ND	ND
IgM^hi^IgD^lo^CD21^hi ^(MZ)	4.4 ± 0.8 (11.2)	4.7 ± 0.5 (16.2)	ND	ND	ND	ND
						
CD3^+^ F4/80^+^	21.3 ± 1.4 0.9 ± 0.1	13.8 ± 0.6 0.9 ± 0.1	15.0 ± 0.7 2.1 ± 1.4	10.4 ± 0.5 3.0 ± 0.7	15.8 ± 1.2 1.1 ± 0.3	23.6 ± 3.3 1.0 ± 0.2
CD94^+^CD3-	1.0 ± 0.3	1.0 ± 0.1	ND	ND	ND	ND
CD16/CD32^+^B220^-^	4.3 ± 1.3	4.4 ± 1.1	ND	ND	ND	ND

Cell numbers are stated at × 10^6^. Values represent the mean ± SD. For B cell subsets, the percentage of each subset in B220^+ ^gated cells is given in parenthesis. AUF1-deficient and wild-type mice used for this analysis were 11-16 weeks of age. ND; not determined.

**Figure 2:**
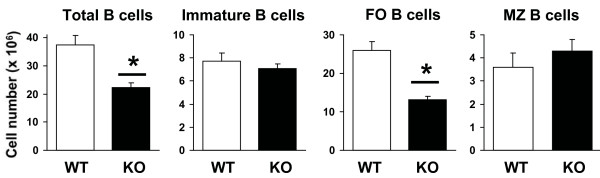
**Splenic B cell populations in AUF1-deficient mice**. Cell populations were determined on the basis of total cell counts and flow cytometric analysis shown in Figure 1. Data are mean ± SD from experiments using eight mice per group. * p < 0.01.

We therefore determined whether the splenocyte loss was restricted to a defined lymphocyte population. The different immature and mature splenic B cell subsets were identified based on the expression of multiple surface markers: CD21, CD23, CD24, IgM, and IgD (Figure 1a) [3]. The analyses presented were performed on mice 11-16 weeks of age, however, older mice analyzed (6 to 9 months of age) showed similar findings to younger mice. This analysis showed that the sharp reduction in splenic B lymphocytes was due predominately to a two-fold reduction in the number of FO B cells (Figure 2). The absolute numbers of transitional (immature) and MZ B cells were unchanged in AUF1^-/- ^mice (Figure 2). Furthermore, analysis of post-transitional precursor subsets, MZ precursor (MZP) B cells and FO-II B cells, showed no differences in absolute numbers (Figure 1b, Table 1). As a result of the decrease in total splenocytes, there was an increase in the percentage of transitional B cells (from 20.6% to 28.0%) and MZ B cells (from 9.6% to 18.4%) whereas, in contrast, there was a decrease in the proportion of mature FO B cells (from 69.3% to 55.4%). As expected with a decrease in splenic FO B cells, there was a decrease in recirculating mature IgM^lo^IgD^hi ^B cells in the bone marrow and lymph node of AUF1-deficient mice (Figure 1a). An analysis of the third mature B cell population, CD5^hi^IgM^hi ^B-1 cells in the peritoneum, showed no differences between wild type and AUFI^-/- ^animals (Figure 1a). All surface markers analyzed were expressed normally in AUF1-deficient splenic B cells, with the exception of CD23, which exhibited reduced expression (Figure 1c). Flow cytometric analysis of CD4, CD8, CD44, and CD62L surface markers of T cells in the spleen showed no differences (Figure 1a), suggesting that the nearly two-fold reduction in splenic T cells was not restricted to a defined T cell population.

Given the specific loss of FO B cells, we examined the expression of AUF1 in different B cell subsets. Surprisingly, flow cytometric analysis demonstrated that AUF1 expression was greatest in wild type MZ and T2 B cells, two populations whose absolute numbers were unaffected in AUF1^-/- ^mice (Figure 1d). AUF1 consists of a family of four protein isoforms that are translated from an alternatively spliced mRNA [27]. Thus, we sorted B cell subsets and then analyzed AUF1 expression by immunoblot analysis to examine individual isoform expression. Consistent with the flow cytometery results, immunoblot analysis demonstrated that AUF1 was most strongly expressed in T2 B cells and MZ B cells. Differences in isoform expression were evident with T2 B cells exhibiting a relative increase in the expression of the p45 isoform. Surprisingly, FO B cells, which were significantly reduced in AUF1-deficient mice, expressed AUF1 at lower levels than other B cell subsets. These findings suggest that a FO B cell-independent process may be responsible for increased loss, such as through regulation of survival factors. Nevertheless, we cannot exclude the possible importance of an altered ratio of AUFI isoform expression, as previously suggested [28].

### Loss of FO B cells is due to absence of AUF1 expression in the hematopoietic lineage

**Figure 3:**
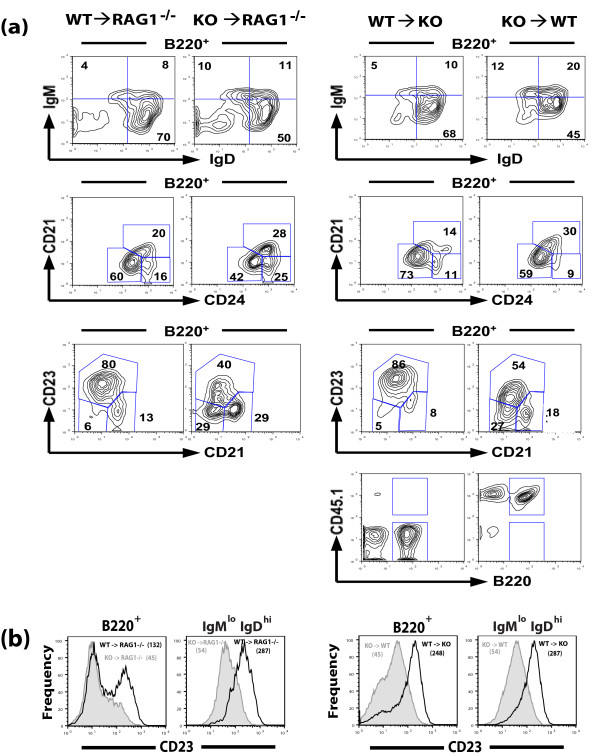
**Flow cytometric analysis of lymphocyte populations in bone marrow chimeric mice**. (a) Ten weeks post bone marrow transplantation, splenocytes from indicated bone marrow chimeric mice were stained with the indicated antibodies and analyzed. Numbers indicate the percentage of lymphoid cells in quadrants or enclosed within indicated gates. Contour plots gated for B220^+ ^are indicated. FO B cells are represented by IgD^hi^IgM^lo^, CD21^int^CD24^lo^, and CD21^int^CD23^hi ^gates and MZ B cells are represented by the CD21^hi^CD23^lo ^gate. Splenocytes in bone marrow chimeras are of donor origin as shown by CD45 analysis. (b) Decreased CD23 expression in B cells (B220^+^) and FO B cells (IgM^lo^IgD^hi ^gated) from chimeric mice receiving bone marrow from AUF1^-/- ^donors. Numbers in parenthesis indicate the MFI of CD23 expression.

To determine whether the differences observed in splenic B cell subsets in AUF1^-/- ^mice were dependent on the expression of AUF1 in hematopoeitic cells, we performed bone marrow adoptive transfer studies. An equal number of bone marrow cells from AUF1-deficient or wild-type mice were transferred into sub-lethally irradiated RAG1^-/- ^mice, which are devoid of mature B and T lymphocytes. Ten weeks after cell transfer, the RAG1^-/- ^recipient mice of AUF1^-/- ^bone marrow, similar to AUF1^-/- ^mice, exhibited a greater than two-fold reduction in number of splenic FO B cells and a sharp decrease in the proportion of FO B cells (from 69.6% to 50.0%) as compared to RAG1^-/- ^recipient mice of wild-type bone marrow (Figure 3a, Table 1). Similarly, irradiated wild-type mice reconstituted with AUF1^-/- ^bone marrow exhibited a reduction in the proportion of FO B cells (from 68.8% to 53.0%) as compared to irradiated AUF1^-/- ^mice reconstituted with wild-type bone marrow (Figure 3a, Table 1). Irradiated RAG1^-/- ^or wild-type recipients of AUF1^-/- ^bone marrow, similar to AUF1^-/- ^mice, showed a reduction in CD23 expression (Figure 3b). These data demonstrate that changes in the frequency of mature B cells and decreased CD23 expression in AUF1^-/- ^mice are intrinsic to the hematopoeitic lineage.

### Decreased number of splenocytes in adult AUF1^-/- ^mice is not rescued by transfer of wild-type bone marrow

**Figure 4:**
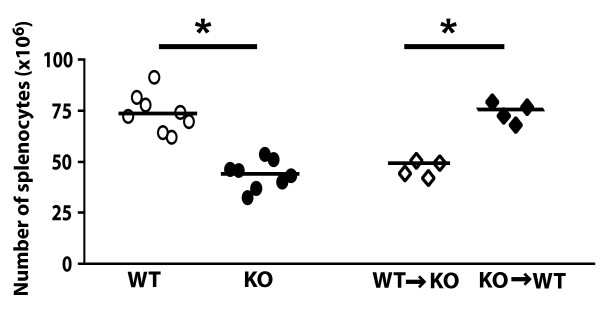
**Number of splenocytes in AUF1^-/- ^mice and bone marrow chimeras**. Each point represents cell counts from a single mouse as determined by hemocytometry. * p < 0.05.

AUF1^-/- ^mice have much smaller spleens that support roughly half the number of splenocytes as compared to wild-type littermates (Figure 4; 70.8 ± 10.7 *vs *41.6 ± 7.6 × 10^6^). To investigate if the decreased cellularity was dependent on AUF1 expression in the hematopoeitic population, bone marrow adoptive transfer experiments were performed. Surprisingly, irradiation of adult AUF1^-/- ^mice and reconstitution with wild-type bone marrow did not rescue the reduction in splenic lymphocyte cellularity (Figure 4). Most simply these adoptive transfer studies suggest that the signals required for determining the number of splenic lymphocytes is likely provided by radiation-resistant stromal cells. However, we cannot rule out the possibility that these signals may be provided by the hematopoeitic lineage in a developmentally-fixed manner, similar to the mechanism described for the lymphotoxin signal needed in proper splenic organization [29,30]. Thus, these bone marrow transfer studies suggest that AUF1 influences two separate processes, since the transfer of wild-type bone marrow cells into AUF1^-/- ^recipients corrects the proportion of FO and MZ B cells, but does not rescue the decreased number of splenocytes (Figure 4; Table 1). Similarly, transfer of AUF1^-/- ^bone marrow cells into wild-type recipients results in a decreased proportion of FO B cells and increased proportion of MZ B cells, but does not alter the absolute number of splenocytes. Thus, we favor the hypothesis that AUF1 influences two separate processes: maintenance of the proportion of specific subsets of the mature B cell population. A function that is autonomous to AUF1 expression; and influencing the overall number of splenocytes, which is not autonomous to AUF1 expression in the hematopoeitic lineage.

### Increased turnover of FO B cells in AUF1-deficient mice

**Figure 5:**
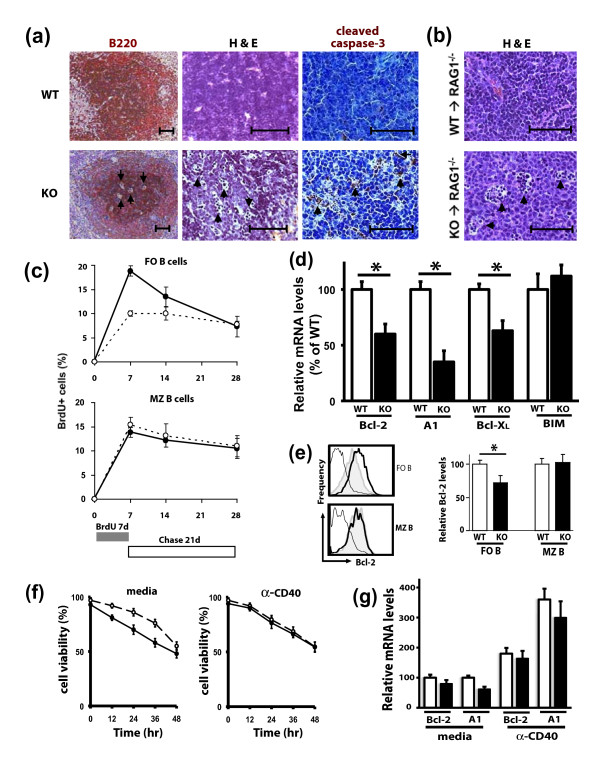
**Prominent apoptosis, increased turnover, and decreased expression of Bcl-2, A1, and Bcl-X_L _in AUF1-deficient FO B cells**. (a-b) Prominent apoptosis (arrows) seen in splenic B cell follicles from (a) AUF1^-/- ^and (b) irradiated RAG1^-/- ^mice reconstituted with AUF1^-/- ^bone marrow. Apoptosis was confirmed by cleaved caspase-3 staining. Scale bar = 0.1 mm. (c) Analysis of *in vivo *B cell turnover. Mice were fed BrdU for 7d, and incorporated BrdU was determined by flow cytometric analysis. The percentage of BrdU-positive FO (CD21^int^CD24^lo^) or MZ (CD21^hi^CD23^lo^) B cells from the spleens of wild-type (white) and AUF1^-/- ^(black) mice was plotted; *n *= 3 per time point. (d) Relative Bcl-2, A1, Bcl-X_L_, BIM mRNA levels in sorted FO B cells from AUF1^-/- ^and wild-type mice were determined by qRT-PCR. Values were normalized to cyclophilin levels and depicted as mean ± SD, *n *= 4. (e) Four-color flow cytometric analysis was performed to identify intracellular Bcl-2 protein levels in FO and MZ B cells. Histograms depict the intensity of wild-type (*bold*) and AUF1^-/- ^(*shaded*) cells stained with Bcl-2 antibody or wild-type stained with isotype-matched control antibody (*thin line*). Quantification of Bcl-2 expression. The normalized values (MFI for Bcl-2 - MFI of isotype-matched control) for AUF1^-/- ^cells (FOB 11.9, MZB 9.6) were plotted as a percentage of wild-type values (FOB 16.9, MZB 9.0). Graph represents mean ± SD, *n *= 4. * p < 0.05. (f-g) Sorted FO B cells were cultured in media alone or with anti-CD40 (10 μg/ml) for times indicated and (f) the percent of live cells was determined by 7-AAD exclusion or (g) Bcl-2 and A1 mRNA levels were determined 24 hrs post culture. Values represent means ± SD for three to four mice in each group.

Mutations affecting either the production or survival of mature B cells can influence the number of B cells in the spleen [31]. Therefore, we next addressed whether the decrease in FO B cells in AUF1^-/- ^mice results from a defect in cell production and/or survival. Examination of B cell follicles in the spleens of AUF1-deficient mice revealed many pyknotic and fragmented cells characteristic of cells undergoing apoptosis (Figure 5a). The striking increase in the presence of apoptotic cells within the splenic follicles of AUF1^-/- ^mice was confirmed by cleaved caspase-3 staining (Figure 5a). Similarly, RAG1^-/- ^mice reconstituted with AUF1^-/- ^bone marrow exhibited pronounced apoptosis in splenic follicles (Figure 5b), a finding not seen in RAG1^-/- ^mice reconstituted with wild-type bone marrow. BrdU pulse-chase studies were performed to directly measure the turnover of FO B cells. Mice were continuously fed BrdU for 7 days and the proportion of BrdU-positive splenic FO B cells (B220^+^CD21^lo^CD24^hi^) was determined by flow cytometery at 0, 7, or 14 days following BrdU administration. At the end of the pulse period, a significantly greater proportion of FO B cells were labeled in AUF1^-/- ^mice as compared to wild-type littermates (Figure 5c). The percentage of BrdU^+ ^FO B cells stayed fairly stable for wild-type control mice during the chase period, but noticeably declined in AUF1^-/- ^mice (Figure 5c), demonstrating a two-fold decrease in the average half-life of AUF1^-/- ^B cells (from 68 to 32 days). In contrast, BrdU pulse-chase studies demonstrated no differences in the labeling or turnover of MZ B cells in AUF1^-/- ^mice (Figure 5c). It is well-established that the expression of Bcl-2 anti-apoptotic and pro-apoptotic family members is vital for the maintenance of B cells within the spleen [32]. Therefore, we assessed the levels of Bcl-2, A1, Bcl-X_L_, and BIM mRNA in freshly sorted FO B cells from AUF1^-/- ^and wild-type mice. AUF1-deficient FO B cells exhibited a significant decrease in all three anti-apoptotic factors (Figure 5d), with a 40% reduction in expression of Bcl-2 and Bcl-X_L _mRNAs and 65% reduction in A1 mRNA levels. However, no significant difference was seen in BIM mRNA levels in FO B cells from AUF1^/- ^and wild-type mice. We further validated these results by comparing Bcl-2 protein levels in mature splenic B cell populations of AUF1^-/- ^mice by flow cytometry. This analysis was restricted to Bcl-2 because it was not possible to measure A1 by flow cytometry. Consistent with a 40% decrease in Bcl-2 mRNA expression, the level of Bcl-2 protein expression was similarly decreased in FO B cells from AUF1^-/- ^mice (Figure 5e). In contrast, MZ B cells from AUF1^-/- ^mice exhibited no difference in Bcl-2 expression as compared to MZ B cells from wild-type littermates (Figure 5e-f). We further characterized FO B cell turnover, by analyzing cell death in cultured cells via 7-AAD analysis. We purified FO B cells based on CD24 and CD21 markers, because purification with IgM and IgD could elicit BCR signals that influence B cell survival. FO B (CD21^int^CD24^lo^) cells lacking AUF1 were more susceptible to spontaneous cell death in the first 36 hours in culture (Figure 5f). CD40 engagement has been shown to be important in the up-regulation of Bcl-2 family members [33]. Thus, FO B cells were stimulated in culture by CD40 engagement to assess the ability to express Bcl-2 family members. AUF1-deficient FO B cells showed no differences in viability or in ability to express anti-apoptotic Bcl-2 family members upon CD40 engagement (Figure 5g). These data indicate that AUF1 deficiency leads to a decrease in FO B cell survival, presumably by impairing the expression of key survival factors Bcl-2, Bcl-X_L_, and A1 in absence of CD40 engagement.

### No defect in BCR signaling, in vitro maturation, or BAFF-R expression in AUF1^-/- ^B cells

**Figure 6:**
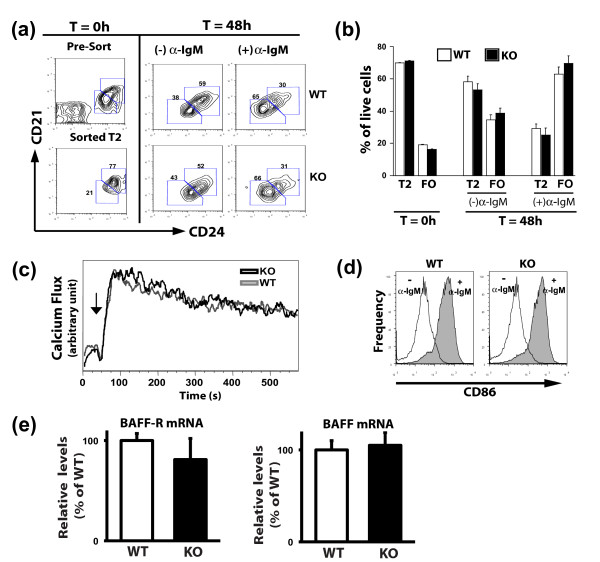
**Normal *in vitro *maturation and BCR signaling in AUF1-deficient B cells**. (a) Sorted T2 B cells (B220^+^CD21^hi^CD24^hi^) were cultured with (+) or without (-) F(ab')_2 _anti-IgM (10 μg/ml) for 48 h, and then analyzed by flow cytometry for CD21 and CD24 expression. (b) The percentage of live cells falling into T2 (CD21^hi^CD24^hi^) and FO (CD21^int^CD24^lo^) gates are shown; mean ± SD, *n *= 3. (c) Calcium flux analysis in response to BCR cross-linking. Arrow indicates time at which anti-IgM (10 μg/ml) was added to cells. (d) CD86 upregulation in B cells with (gray) or without (white) stimulation with F(ab')_2 _anti-IgM. Data in (c-d) is representative of three independent experiments. (e) mRNA levels of BAFF-R in sorted FO B cells (*left panel*) and BAFF in splenocytes (*right panel*) as determined by qRT-PCR.

To directly determine whether AUF1 plays a role in the development of FO B cells, we assayed the *in vitro *maturation of T2 B to FO B cells, as previously described [9]. BCR engagement on T2 B cells drives their differentiation to a mature B cell phenotype, as characterized by down-regulation of CD21 and CD24 surface expression. B220^+^CD21^hi^CD24^hi ^T2 splenocytes were sorted and cultured in the presence or absence of BCR stimulation and analyzed after 48 h (Figure 6a-b). Consistent with the BrdU studies, AUF1^-/- ^T2 B cells exhibited no defect in maturation to a mature B cell phenotype *in vitro *(Figure 6b), suggesting that the BCR signaling pathway mediating this transition is unaffected by the absence of AUF1. AUF1^-/- ^B cells showed no differences in the duration or amplitude of Ca^2+ ^flux (Figure 6c), cell size increase (data not shown), and up-regulation of the activation marker CD86 (Figure 6d) post-BCR ligation, as compared to wild-type B cells. These results therefore indicate that the proximal events of BCR-mediated signaling, shown to be vital in B cell maturation and survival [13,16], are unaffected in AUF1^-/- ^B cells. BAFF, a member of the TNF superfamily, and its major receptor BAFF-R have been shown to be critical in supporting transitional B cell maturation and enhancing the survival of mature B cells [34,35]. No differences were observed in the mRNA levels of BAFF-R in sorted FO AUF1^-/- ^B cells or in BAFF mRNA in AUF1^-/- ^spleens as compared to wild-type controls (Figure 6e).

### Decreased T cell-independent humoral response in AUF1^-/- ^mice

**Figure 7:**
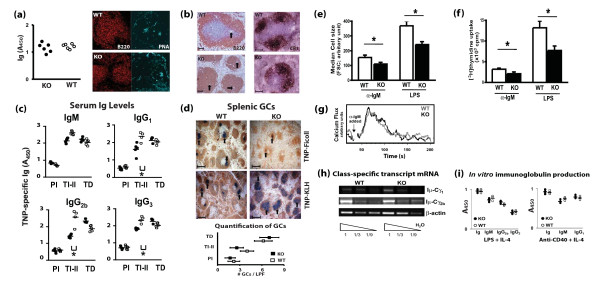
**Impaired T-cell independent humoral response in AUF1^-/- ^mice**. (a) Total serum immunoglobulin levels in naïve mice. Each point represents the value obtained from an individual mouse, AUF1^-/- ^(black) or wild-type (white); *n *= 6. Naïve spleens were stained for B cells (B220) and germinal centers (PNA). (b) B cell follicles were visualized by antibody to B220 and arrows indicate splenic arterioles. Follicular dendritic cells were visualized by antibody to CR1. Scale bar = 0.1 mm. (c) Serum immunoglobulin (Ig) levels in naïve mice (preimmune, PI), and in mice post immunization with TNP-Ficoll (TI-II) or TNP-KLH (TD). Each point represents the value obtained from an individual mouse, AUF1^-/- ^(black) or wild-type (white); *n *= 5, * p < 0.05. (d) Spleen sections from immunized wild-type and AUF1^-/- ^mice were stained for B cells with anti-B220 (brown) and peanut agglutinin (PNA; blue) to visualize germinal centers (GCs, arrows). Scale bar = 0.5 mm. Numbers of GCs per lower power field (LPF, 10×) were quantified. Values represent means from 5 mice (5 fields per mouse) ± SD. (e) Median cell size and (f) proliferation response to anti-IgM (10 μg/ml) or LPS (10 μg/ml) in sorted MZ B cells. Plots represent mean ± SD, *n *= 3; * p < 0.05. (g) Calcium flux analysis in response to BCR cross-linking. Arrow indicates time at which anti-IgM (10 μg/ml) was added to cells. Data is representative of three independent experiments. (h) RT-PCR directed at amplifying post-switch targets composed of Iμ exon spliced onto the 5' exon of Cγ_1 _and Cγ_2b _gene or β-actin gene control performed on B cells stimulated with LPS + IL-4 for 4 days. (i) Total secreted immunoglobulin (Ig) levels or indicated isotypes in culture supernatants from B cells stimulated with LPS + IL-4 or anti-CD40 + IL-4 for 4 days as determined by ELISA.

To determine the functional consequence of AUF1 deficiency, we next measured the ability of AUF1^-/- ^mice to mount a normal humoral immune response to antigenic challenge. TI antigen, TNP-Ficoll, or TD antigen, TNP-KLH, was injected intraperitoneally into AUF1^-/- ^and wild-type mice. Serum levels of TNP-specific antibodies were measured 7 and 14 days post-immunization with TNP-Ficoll and TNP-KLH, respectively. In the absence of antigenic challenge, we observed no differences in basal serum Ig levels between AUF1^-/- ^and wild-type mice (Figure 7a, 7c). Consistent with this finding, we observed no differences in architecture or number of germinal centers (Figure 7a, 7d) or follicular dendritic network (Figure 7b) in spleens of AUF1^-/- ^mice. However, AUF1^-/- ^spleens showed distortion of distinct B and T cell zones, as B cells are seen in PALS, a T cell area largely devoid of B cells in spleens from wild-type mice (Figure 7b). AUF1^-/- ^mice mounted a robust TD humoral response to immunization with TNP-KLH, consistent with the presence of very large germinal centers in the center of B cell follicles, where T cells normally reside (Figure 7d). Given the decrease in the number of FO B cells, one might have expected attenuation rather than augmentation of the TD humoral response. This discrepancy may stem from organizational defects that lead to aberrant germinal centers in the PALS in AUF1^-/- ^mice. It may also reflect the increased T cell response as a result of aberrant control of IL-2 in AUF1^-/- ^mice [36]. In contrast, AUF1^-/- ^mice showed a decreased humoral response to a TI antigen, TNP-Ficoll (Figure 7a). Given the importance of MZ B cells in an effective TI humoral response [37], we cannot rule out that AUF1 is important for proper MZ B cell function, especially given the high relative expression of AUF1 in this population (Figure 1d). This is supported by attenuation of cell size increase and attenuated proliferative response to BCR-ligation and LPS in AUF1-deficient MZ B cells as compared to wild-type controls (Figure 7e-f). There was no difference in calcium flux post-BCR ligation in MZB cells (Figure 7g), which suggests that the impact of AUF1 on MZ B cell function is downstream of calcium mobilization.

It has been suggested that as part of the LR1 transcription complex, AUF1 may play a role in heavy chain class switch recombination (CSR) [38]. Isotype CSR requires the transcription of class-specific mRNAs [39]. RT-PCR directed at amplifying post-switch targets composed of the I_μ _exon spliced onto the 5' exon of the Cγ_1 _and Cγ_2b _gene, loci reported to bind LR1 with the highest affinity [40], was performed on purified cultured B cells 4 days post-activation. AUF1^-/- ^B cells did not display any defect in class-specific transcripts (Figure 7h). Furthermore, AUF1^-/- ^B cells cultured in the presence of IL-4 and either LPS or an antibody to CD40 exhibited no defect in the *in vitro *production of serum Igs (Figure 7i), indicating that AUF1 does not play a direct role in CSR activity in B cells. However, we cannot rule out that AUF1 may play an indirect role in proper humoral responses *in vivo *through the regulation of key cytokines important in CSR and immunoglobulin production.

## Discussion

Our results show that AUF1 deficiency leads to a disturbance in mature splenic B cell populations and in humoral immune responses. Past studies of mice with defective BCR signaling have demonstrated that the strength of the BCR signal is important in both maturation and determination of the cell-fate of the immature B cells [2,11,15]. Furthermore, these studies collectively indicate that weak BCR signals favor MZ B cell development, whereas relatively strong signals favor the development of FO B cells, and perhaps even stronger signals favor the generation of B1 cells [11]. Our results indicate that the reduction of B cell numbers is specific to FO B cells, as normal numbers of T1, T2, FO-II, MZP, and MZ B cells are seen in spleens of AUF1^-/- ^mice, as well as in irradiated RAG1^-/- ^mice reconstituted with AUF1^-/- ^bone marrow. These data suggest that there is no developmental block, nor are transitional B cells diverted to the mature MZ B cell population at the expense of FO B cells, in contrast to mice with impaired BCR signaling [11]. In agreement with this hypothesis, there is no impairment in production of FO B cells *in vitro *as shown by maturation assays, or *in vivo *as demonstrated by BrdU labeling studies. Overall, we conclude that development of FO B cells is unaffected in AUF1^-/- ^mice. In addition to its role in development, signals processed by the BCR complex are required for mature B cells to survive in the periphery. The specific loss of FO B cells in AUF1^-/- ^mice does not seem to result from abnormal BCR signaling, as downstream events of BCR engagement, such as calcium mobilization are unaffected in AUF1^-/- ^B cells.

The phenotype seen in AUF1^-/- ^mice does not seem to involve BAFF signaling, which has been shown to be important in both B cell development and homeostasis [34,35]. BAFF, a member of the TNF superfamily, and its receptors, BR3 and TACI, are not encoded by ARE-mRNAs and are therefore unlikely to be direct targets of AUF1. As predicted, no difference was observed in the expression of BAFF or its receptor in AUF1-deficient mice. Furthermore, the phenotype of BAFF-deficient mice differs from that of AUF1^-/- ^mice. BAFF-deficient mice exhibit a loss of both MZ and FO B cells, as well as severely reduced antibody titers in response to both TD and TI antigens [34,41]. In contrast, AUF1^-/- ^mice exhibit a selective loss of the FO B cell subset and a moderate attenuation of the TI humoral response. BAFF signaling is important in CD21 expression in B cells independent of its role in cell survival [41], and its regulation of CD23 expression is less clear. There are conflicting reports on the role of BAFF on CD23 expression from BAFF-deficient, BAFF-R-deficient, and transgenic BAFF mice [41-43]. In contrast, AUF1-deficient B cells show normal CD21 surface expression, but exhibit a reduction in CD23 surface expression (Figure 1). Although direct functions of AUF1 cannot be ruled out, alterations in local factors such as interleukin-4 in part may explain the decrease in CD23 expression in AUF1^-/- ^B cells [44]. CD23 expression on B cells is involved in antigen presentation and IgE response, as demonstrated by the enhanced IgE response elicited in CD23-deficent mice [45]. We have previously shown that AUF1^-/- ^mice have increased circulating IgE levels [36] that may be due to the reduced CD23 expression on AUF1-deficient B cells.

In the periphery, a lymphocyte acquires survival signals through receptors for cytokines, antigens, and hormones [8,46]. Failure to adequately acquire such signals results in cell death and thus determines the size of the peripheral lymphocyte pools [8,46,47]. Although signaling through the BCR is required for the maintenance of mature splenic B cells [9], it is not the only requirement. The splenic microenvironment also plays an essential role in the maintenance of mature B cells [17]. However, knowledge regarding the nature and regulation of the splenic microenvironment is limited, particularly as it pertains to the accumulation and maintenance of splenic lymphocytes. Our work suggests that AUF1 is important in the regulation of survival signals needed for the maintenance of FO B cells within the spleen, as indicated by decreased Bcl-2, A1, and Bcl-X_L _levels. Spleens from AUF1^-/- ^mice bear a strong resemblance to those of Bcl-2-deficient mice in that they are smaller, show a loss of mature B and T cells, and show many apoptotic cells [32]. Mature recirculating IgM^lo^IgD^hi ^FO B cells from lymph nodes of AUF1^-/- ^mice exhibit no significant differences in Bcl-2 expression (see Additional file 1), suggesting that the survival signal provided in lymph nodes is unaffected by the absence of AUF1. These findings highlight differences in survival signals necessary for recirculating mature B cells in different lymphoid organs. In support of this hypothesis, it has been recently shown that macrophage migration inhibitory factor (MIF), through the activation of the CD74-CD44 complex, is necessary in the maintenance of mature B cells in the bone marrow, but not in the spleen or lymph nodes [48]. The spleen-specific alterations in lymphocyte populations in AUF1^-/- ^mice suggests that AUF1 acts in regulating the local microenvironment, as opposed to regulating global factors in lymphocyte development, such as BCR signaling that appears normal in AUF1^-/- ^B lymphocytes. Future studies with a more comprehensive screening approach, such as cDNA micro-arrays, may prove valuable in identifying mRNAs regulated by AUF1 that play a role in FO B cell lymphocyte survival within the spleen. CD40 engagement on mature B cells results in induction of anti-apoptotic Bcl-2 family members, which protect these cells from antigen receptor-mediated apoptosis [33] and are required for GC formation, progression [49] and TD response [50]. AUF1^-/- ^FO B cells were able to appropriately induce the expression of Bcl-2 and A1 in response to CD40 engagement, which supports their ability to form germinal centers in AUF1-deficient mice.

An important phenotype of AUF1-deficiency is a decrease in the number of splenic lymphocytes and a skewing of the B cell subpopulations, with an over-representation of immature and MZ B cells and a decrease in FO B cells. There are several possible mechanisms that may account for this complex phenotype. AUF1 may regulate a survival signal needed for the maintenance of FO B cells, the largest component of splenic B cells, and loss of this population could lead to a decrease in the accumulation of T cells within the spleen. Previous studies on B cell-deficient mice have reported a decrease in splenic T cell numbers [30,51]. BCR-deficient mice exhibit a three-fold reduction in the number of splenic CD4 and CD8 T cells, although no loss of T cells was seen in lymph nodes from these animals [30]. These results suggest that B cells provide signals that promote CD4 and CD8 T cell accumulation and/or survival in the spleen [30]. Notably, CD3ε- and CD4-deficient mice show a 40% reduction of splenic mature B cells, suggesting that T cells may also impact the survival of B cells within the spleen [31]. Likewise, AUF1 may regulate survival signals required independently for the maintenance of FO B and T cells. At this time we cannot distinguish whether the loss of T and B cells within the spleen are related, or whether both populations, independent of each other, are deficient in survival signals, resulting in decreased Bcl-2 expression in both populations in AUF1^-/- ^mice (see Additional file 1). These mechanisms predict that the 50% decrease in splenic B cells in AUF1^-/- ^mice results from increased FO B cell turnover that outpaces the rate of FO B cell production by two-fold. However, BrdU studies indicate that there is a 2.1-fold decrease in splenic FO B cell average half-life and a 1.7-fold increase of B cell production in AUF1^-/- ^mice. This would result in a 30% decrease in FO B cells and not the 50% decrease observed in AUF1^-/- ^mice. Furthermore, if turnover did outpace production by two-fold, one would predict a greater decrease than the 20% seen in recirculating mature B cells in lymph nodes and bone marrow of AUF1^-/- ^mice.

The data from bone marrow adoptive transfer studies more likely indicate that two separate processes are involved in the observed phenotype: (1) AUF1 is important in the maintenance of FO B cells within the spleen, most likely through regulation of survival factors; and (2) AUF1 impacts the number of lymphocytes within the spleen through the regulation of yet unknown signals. Adoptive transfer studies support the involvement of two separate processes, as reconstitution of irradiated AUF1^-/- ^mice with wild-type bone marrow corrects the shifts in B cell subpopulation proportions but not the overall number of splenocytes. The involvement of two separate processes also better explains the deficit in FO B cells. The 40% decrease in the number of splenocytes, in addition to the increased FO B cell turnover that would result in a 30% decrease in the proportion of FO B cells, more accurately approximates the 50% decrease in the number of FO B cells observed in AUF1^-/- ^mice.

The complex phenotype seen in AUF1-deficient mice is not surprising, given that AUF1 regulates the expression of numerous immune-modulating cytokines and chemokines [20,36,52,53]. Although we have focused here on defects in the B cell compartment in AUF1-deficient mice, we have previously shown defects in the T cell and macrophage compartments [36]. Moreover, despite the fact that the FDC network can be visualized, we cannot exclude that the dendritic subset may be affected in AUF1-deficient mice and may impact the function, size, and location of other immune cell compartments. Tristetraprolin, another ARE-binding protein, binds and regulates the expression of mRNAs encoding key regulators of human dendritic cell maturation [54]. Future analysis of specific targets of AUF1 in different immune cell subsets will be important in dissecting the role of AUF1 on individual populations leading to the overall complex phenotype seen in its deficiency in the mouse model.

## Conclusion

This work demonstrates that AUF1 is important in determining the size of the splenic lymphocyte population and proper survival of FO B cells within the spleen. Future studies will address the identification and regulation of AUF1 targets as they pertain to splenic size, FO B cell maintenance, and humoral immune response.

## Abbreviations

7-AAD: 7-amino-actinomycin D; ARE: adenosine/uridine-rich element; BCR: B cell receptor; BAFF: B cell activating factor; FO: follicular; GC: germinal center; MFI: mean fluorescence intensity; MZ: marginal zone; PALS: periarteriolar lymphoid sheath; T1: transitional type-1; T2: transitional type-2; TI: T-cell independent; TD: T-cell dependent.

## Authors' contributions

NS made substantial contributions to conception and design of the study, acquired, analyzed, and interpreted the data, and drafted the manuscript. RJS contributed in data analysis and writing of the manuscript. JL and MB made contributions to the study design and acquisition of data. All authors read and approved the final manuscript.

## Authors' information

The authors declare no competing financial interests. Correspondence and requests for materials should be addressed to R.J.S. (Robert.Schneider@nyumc.org).

## Supplementary Material

Additional file 1 **Bcl-2 expression in T and FO B cells from spleens and lymph nodes from AUF1^-/- ^mice**. Intracellular Bcl-2 protein levels in FO B, CD4^+ ^T, and CD8^+ ^T cells from spleens and lymph nodes were determined by four-color flow cytometric analysis. Histograms depict the intensity of wild-type (bold) and AUF1^-/- ^(shaded) cells stained with Bcl-2 antibody or wild-type stained with isotype-matched control antibody (thin line). Bcl-2 expression was quantified. The normalized values (mean fluorescence intensity (MFI) for Bcl-2 - MFI of isotype-matched control) for AUF1^-/- ^cells were plotted as a percentage of wild-type values. Plot represents mean ± SD for four mice in each group. * p < 0.05. Click here for file
